# Analysis of Initiating Anticoagulant Therapy for Atrial Fibrillation Among Persons Experiencing Homelessness in the Veterans Affairs Health System

**DOI:** 10.1001/jamanetworkopen.2022.23815

**Published:** 2022-07-26

**Authors:** David A. Wilson, Osei Boadu, Audrey L. Jones, Nadejda Kim, Maria K. Mor, Leslie R. M. Hausmann, Utibe R. Essien

**Affiliations:** 1 Center for Health Equity Research and Promotion, VA Pittsburgh Healthcare System, Pittsburgh, Pennsylvania; 2 Department of Medicine, University of Pittsburgh School of Medicine, Pittsburgh, Pennsylvania; 3 Informatics, Decision-Enhancement and Analytic Sciences Center, VA Salt Lake City Health Care System, Salt Lake City, Utah; 4 Department of Internal Medicine, University of Utah School of Medicine, Salt Lake City; 5 Department of Biostatistics, University of Pittsburgh Graduate School of Public Health, Pittsburgh, Pennsylvania

## Abstract

This cohort study examines the use of anticoagulant therapy among persons who have experienced homelessness treated for atrial fibrillation in the Veterans Affairs Health System.

## Introduction

In the US, more than 37 000 veterans are homeless every night.^1^ Persons who have experienced homelessness (PEH) have a higher burden of cardiovascular diseases, such as atrial fibrillation (AF),^2^ documented challenges accessing health care, and suboptimal management of cardiovascular conditions.^3^ Stroke-preventing anticoagulant therapy improves AF outcomes, but its use among PEH is unknown.

## Methods

This cohort study used data from the Race, Ethnicity, and Anticoagulation Choice in Atrial Fibrillation (REACH-AF) cohort to compare rates and types of anticoagulant therapy among PEH vs non-PEH.^4^ Using administrative and clinical data from the Veterans Health Administration (VA), we defined the REACH-AF cohort as patients with a new AF diagnosis between January 1, 2010, and December 31, 2020, continuous VA enrollment for 2 years before diagnosis, and an outpatient confirmatory AF diagnosis within 180 days of the index diagnosis (eFigure in the Supplement). We excluded patients with valvular heart disease, cardiac ablation, hyperthyroidism, an anticoagulant prescription in the 2 years before the index diagnosis, or death or hospice care in the 90 days after diagnosis. The institutional review board at the VA Pittsburgh Healthcare System approved the study and granted a waiver of informed consent owing to use of deidentified data. We followed the STROBE reporting guideline.

Our primary outcome was anticoagulant therapy initiation, defined as the first outpatient prescription for any anticoagulant within 90 days of the index diagnosis. Among individuals initiating anticoagulant therapy, we determined whether patients were prescribed warfarin or a direct oral anticoagulant (DOAC). Our independent variable was homeless experience documented with *International Classification of Diseases, Ninth Revision*, or *International Statistical Classification of Diseases and Related Health Problems, Tenth Revision* codes, receipt of VA homeless services, or a positive screen on the VA annual homeless screener (eTable in the Supplement) in the year before the index diagnosis.^5^ We estimated differences in anticoagulant initiation, then type of therapy, using mixed-effects logistic regression models adjusted for sociodemographic characteristics, including self-reported race and ethnicity,^4^ clinical, and clinician and facility covariates. We included a random effect for VA site in all models and used a threshold of *P* < .05 (2-tailed) for statistical significance. Data analysis was performed using SAS Enterprise Guide, version 8.2 (SAS Institute Inc).

## Results

Among 168 003 patients with incident AF from 2014 to 2020, 164 396 were men (97.9%), 3607 were women (2.1%), with a mean (SD) age of 66.0 (10.6) years; 6362 (3.8%) had experiences of homelessness (Table). Anticoagulant initiation was lower among PEH (3576 [56.2%]) than non-PEH (106 265 [65.7%]) (*P* < .001). In our final adjusted model, the odds of initiating any anticoagulant were significantly lower for PEH (adjusted odds ratio [aOR], 0.79; 95% CI, 0.74-0.84) (Figure). Among anticoagulant initiators, DOAC use appeared to be lower for PEH (2514 [70.3%]) than for non-PEH (79 691 [75.0%]) (*P* < .001). However, these differences were not statistically significant in the final adjusted model (aOR, 0.96; 95% CI, 0.87-1.06).

**Table.** Characteristics of Patients With and Without Homeless Experience, Among Veterans With Incident Atrial Fibrillation^a^

Variable	No. (%)		*P* value
	Homeless (n = 6362)	Nonhomeless (n = 161 641)	
**Sociodemographic characteristics**			
Age at diagnosis, y			
≤64	2882 (45.3)	25 796 (16.0)	<.001
65-74	2276 (35.8)	68 741 (42.5)	
75-84	794 (12.5)	42 387 (26.2)	
≥85	410 (6.4)	24 717 (15.3)	
Sex			
Male	6157 (96.8)	158 239 (97.9)	<.001
Female	205 (3.2)	3402 (2.1)	
Race and ethnicity			
American Indian-Alaska Native	63 (1.0)	792 (0.5)	<.001
Asian	68 (1.1)	1902 (1.2)	
Non-Hispanic Black	1531 (24.1)	14 218 (8.8)	
Hispanic	274 (4.3)	5774 (3.6)	
Multiple	69 (1.1)	878 (0.5)	
Non-Hispanic White	4318 (67.9)	137 446 (85.0)	
Region			
Midwest	1259 (19.8)	40 195 (24.9)	<.001
Northeast	949 (14.9)	24 379 (15.1)	
South	2254 (35.4)	64 626 (40.0)	
West	1830 (28.8)	29 286 (18.1)	
US territories^b^	19 (0.3)	1019 (0.6)	
Rurality			
Large metropolitan	3415 (53.7)	65 162 (40.3)	<.001
Small metropolitan	2104 (33.1)	57 684 (35.7)	
Micropolitan	501 (7.9)	20 335 (12.6)	
Rural	290 (4.6)	16 296 (10.1)	
VA enrollment priority group^c^			
1-3	2367 (37.2)	75 611 (46.8)	<.001
4	249 (3.9)	3481 (2.2)	
5	3029 (47.6)	37 756 (23.4)	
6	52 (0.8)	5435 (3.4)	
7-8	415 (6.5)	34 829 (21.5)	
Year			
2014	744 (11.7)	19 680 (12.2)	.003
2015	843 (13.3)	21 671 (13.4)	
2016	847 (13.3)	23 337 (14.4)	
2017	991 (15.6)	25 274 (15.6)	
2018	1058 (16.6)	27 205 (16.8)	
2019	1076 (16.9)	26 510 (16.4)	
2020	803 (12.6)	17 964 (11.1)	
**Clinical characteristics**			
Selected medical comorbidities			
Congestive heart failure	1657 (26.0)	26 951 (16.7)	<.001
Hypertension	4874 (76.6)	121 672 (75.3)	.02
Diabetes	2448 (38.5)	61 044 (37.8)	.25
Vascular disease	2290 (36.0)	51 936 (32.1)	<.001
Prior stroke	1129 (17.7)	20 942 (13.0)	<.001
Prior bleeding	1833 (28.8)	36 139 (22.4)	<.001
Liver disease	739 (11.6)	7928 (4.9)	<.001
Kidney disease	1171 (18.4)	18 905 (11.7)	<.001
Mental health	4002 (62.9)	47 608 (29.5)	<.001
Substance use disorder	2910 (45.7)	22 838 (14.1)	<.001
Medications predisposing to bleeding	4404 (69.2)	77 288 (47.8)	<.001
Oral anticoagulant use			
Warfarin	1062 (16.7)	26 576 (16.4)	<.001
Direct oral anticoagulant	2514 (39.5)	79 691 (49.3)	<.001
None	2786 (43.8)	55 376 (34.3)	<.001
Body mass index^d^			
<18.5	131 (2.1)	1629 (1.0)	<.001
18.5 to <25.0	1512 (23.8)	30 050 (18.6)	
25.0 to <30.0	1865 (29.3)	54 035 (33.4)	
30.0 to <35.0	1358 (21.3)	40 792 (25.2)	
35.0 to <40.0	786 (12.4)	20 179 (12.5)	
≥40.0	597 (9.4)	12 439 (7.7)	
CHA_2_DS_2_VASc stroke risk (score)^e^			
Low (0-1)	1515 (23.8)	23 834 (14.7)	<.001
Moderate (2-4)	3627 (57.0)	106 115 (65.6)	
High (>4)	1220 (19.2)	31 692 (19.6)	
Frailty index, mean (SD)			
Nonfrail (≤0.1)	1454 (22.9)	58 643 (36.3)	<.001
Prefrail (>0.1-0.2)	1830 (28.8)	54 419 (33.7)	
Mildly frail (>0.2-0.3)	1493 (23.5)	29 612 (18.3)	
Moderately frail (>0.3-0.4)	899 (14.1)	12 457 (7.7)	
Severely frail (>0.4)	686 (10.8)	6510 (4.0)	
**Clinician/facility characteristics**			
Specialty of initial AF-diagnosing clinician			
Cardiology	1011 (15.9)	22 286 (13.8)	<.001
Emergency department	1271 (20.0)	21 506 (13.3)	
Primary care	2633 (41.4)	83 985 (52.0)	
Pharmacy	845 (13.3)	23 892 (14.8)	
Other	602 (9.5)	9972 (6.2)	
Facility type			
VAMC	4922 (77.4)	105 456 (65.2)	<.001
Primary care CBOC	561 (8.8)	24 416 (15.1)	
Multispecialty CBOC	641 (10.1)	23 246 (14.4)	
Other	214 (3.4)	8494 (5.3)	
Seen by cardiology within past 90 d of AF	3782 (59.4)	80 158 (49.6)	<.001
Seen by anticoagulant clinic within past 90 d of AF	1335 (21.0)	36 722 (22.7)	.001
≥2 PC visits in the past year	5376 (84.5)	122 071 (75.5)	<.001

Abbreviations: AF atrial fibrillation; CBOC, community-based outpatient clinic; PC, primary care; VA, Veterans Health Administration; VAMC, Veteran Affairs medical center.

^a^ All percentages were calculated with missing data removed from the denominator. Data were missing for less than 5% of patients for region, rurality, area deprivation index, VA enrollment priority group, body mass index, and facility type and less than 0.5% for the remaining variables.

^b^ US territories such as Guam, American Samoa, and Puerto Rico.

^c^ Priority groups convey veterans’ level of eligibility for VA services; lower-level groups have increased eligibility.

^d^ Calculated as weight in kilograms divided by height in meters squared.

^e^ CHA_2_DS_2_VASc indicates a score composed of points for congestive heart failure; hypertension; age greater than or equal to 75 years; diabetes; prior stroke, transient ischemic attack, or thromboembolism; vascular disease; age 65 to 74 years; and sex category (female).

**Figure.**
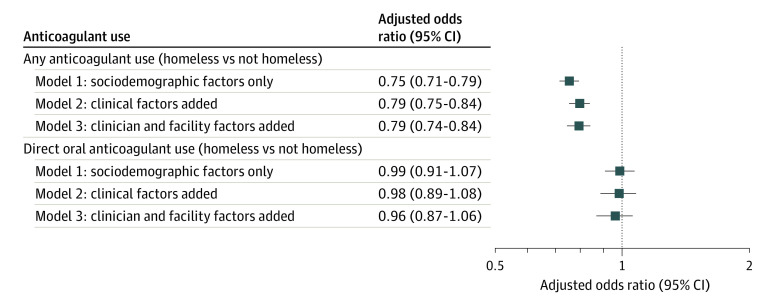
Adjusted Odds of Initiating Any Anticoagulant and Direct Oral Anticoagulant Therapy vs Warfarin Among Patients With and Without Experiences of Homelessness In mixed-effects logistic regression models adjusting for patient sociodemographic (model 1), clinical factors (model 2), and facility and clinician factors (model 3), there were significant differences in any anticoagulant initiation with lower initiation among persons experiencing homelessness. Among patients who initiated therapy, there was no significant difference in the type of therapy used by patients with and without experiences of homelessness.

## Discussion

In a national cohort of veterans with AF, we found substantial disparities in anticoagulant therapy prescribing between PEH and non-PEH. Among those who initiated therapy, however, there was no significant difference in DOAC use. The limitations include the use of a VA cohort that may affect generalizability to a broader population and the inability to assess prescription copayment and other social barriers to pharmacotherapy access. Furthermore, we were unable to assess the duration of homelessness or exclude residual confounding. To our knowledge, this is the first report observing national differences in anticoagulation among PEH with AF in the DOAC era.^6^ Our findings suggest that efforts to prevent stroke and improve AF management among PEH may best focus on addressing treatment initiation barriers.

That these findings persisted after adjusting for sociodemographic and clinical factors within an integrated health system with a low-cost, uniform drug formulary and integrated medical and social services has implications for equitable AF management. Research into the determinants of these observed inequities, including clinician bias, determination of VA priority groups, and differential shared decision-making among high-risk populations will be critical for improving the quality of AF care.

## Notes

Supplement. **eTable.** Identification of Homelessness by Diagnosis and Clinical Codes and Screening Questions **eFigure.** Study Flow Diagram
