# Supplementary material for: Analysis of Initiating Anticoagulant Therapy for Atrial Fibrillation Among Persons Experiencing Homelessness in the Veterans Affairs Health System
Source: JAMA Netw Open. 2022 Jul 26;5(7):e2223815. doi: 10.1001/jamanetworkopen.2022.23815 (PMC9327581; doi:10.1001/jamanetworkopen.2022.23815)
Supplement: Supplement. — eTable. Identification of Homelessness by Diagnosis and Clinical Codes and Screening Questions eFigure. Study Flow Diagram [file jamanetwopen-e2223815-s001.pdf]

Supplementary Online Content

Wilson DA, Boadu O, Jones AL, et al. Analysis of initiating anticoagulant therapy for atrial fibrillation among persons experiencing homelessness in the Veterans Affairs health system. *JAMA Netw Open*. 2022;5(7):e2223815. doi:10.1001/jamanetworkopen.2022.23815

eTable. Identification of Homelessness by Diagnosis and Clinical Codes and Screening Questions

eFigure. Study Flow Diagram

This supplementary material has been provided by the authors to give readers additional information about their work.

© 2022 Wilson DA et al. *JAMA Network Open.*

eTable. Identification of Homelessness by Diagnosis and Clinical Codes and Screening Questions

ICD-9 Code[1]	ICD Code Descriptions
V60.0	Lack of housing
V60.1	Inadequate housing
V60.89	Other specified housing or economic circumstances
V60.9	Unspecified housing or economic circumstance
**ICD-10 Code[1]**	**ICD Code Descriptions**
Z59.0	Homelessness
Z59.1	Inadequate housing
Z59.8	Other problems related to housing and economic circumstances
Z59.9	Problem related to housing and economic circumstances, unspecified
**VA Clinical Code**	**Clinical Code Descriptions**
28[1]	Mental Health Residential and Rehab Treatment Program for Compensated Work Therapy/Treatment Resident (MH RRTP CWT/TR)
37[1]	Domiciliary care for homeless Veterans (DCHV)
504[2]	Services provided by VA clinical staff of Grant and Per Diem programs to homeless vets or family members of such vets (Group Assistance)
507[2]	Services provided by VA for HUD-VASH to homeless or at-risk Veterans or family members (Group Assistance)
508[2]	HCHV/HCMI Group (VA Health Care for Homeless Veterans/Homeless Chronically Ill)
511[2]	Services provided by VA clinical staff to Homeless Veterans or family members of such who are receiving services from Grant or per Diem programs
522[1,2]	HUD/VASH (Dept. of Housing and Urban Development VA Shared Housing) Individual
528[1,2]	Telephone Services/Homeless Mentally Ill (HMI)
529[1,2]	Healthcare for homeless Veterans (restricted to programs approved by the Northeast Program Evaluation Center, NEPEC)
530[1]	Telephone/HUD-VASH (Dept. of Housing and Urban Development VA Shared Housing)
555[2]	Homeless Veteran Community Employment services – Individual Assistance
556[2]	Homeless Veteran Community Employment services – Group Assistance
590[1,2]	Community outreach to homeless Veterans

Homeless Screening Questions[3]	Positive Indicator
In the past 2 months, have you been living in stable housing that you own, rent, or stay in as part of a household?	If no: Has no stable housing
Are you worried or concerned that in the next 2 months you may not have stable housing that you own, rent, or stay in as part of a household?	If yes: Has worries about stable housing

[1] Peterson R, Gundlapalli AV, Metraux S, et al. Identifying homelessness among veterans using VA administrative data: opportunities to expand detection criteria. *PLoS One.* 2015;10(7):e0132664.

[2] Garvin LA, Hu J, Slightam C, McInnes DK, Zulman DM. Use of video telehealth tablets to increase access for veterans experiencing homelessness. *J Gen Intern Med*. 2021 Aug;36(8):2274-2282. doi: 10.1007/s11606-021-06900-8.

[3] Montgomery AE, Rahman AKMF, Chhabra M, Cusack MC, True JG. The importance of context: linking veteran outpatients screening positive for housing instability with responsive interventions. *Adm Policy Ment Health*. 2021;48(1):23-35.

© 2022 Wilson DA et al. *JAMA Network Open.*

eFigure. Study Flow Diagram. Among 992,453 patients in the Veterans Health Administration (VA) with an incident diagnosis of atrial fibrillation (AF) from 2010 through 2020, we identified 325,984 with continuous VA enrollment, no prior diagnosis of AF and a confirmatory AF diagnosis within 180 days after the index diagnosis in VA. After additional exclusions, the final study sample included 168,003 patients with incident AF from 2014-2020. All exclusions were performed sequentially.

© 2022 Wilson DA et al. *JAMA Network Open.*

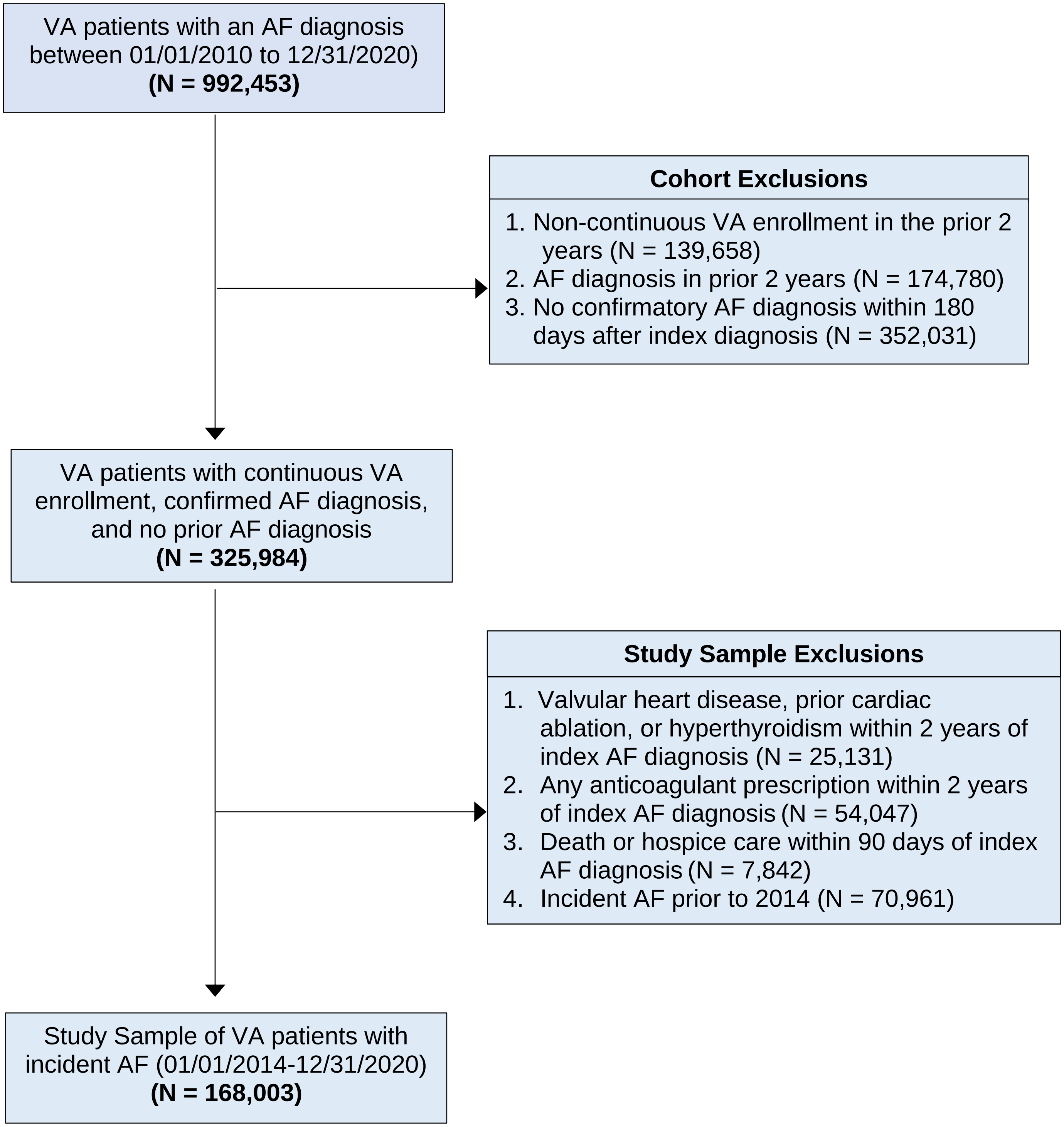

© 2022 Wilson DA et al. *JAMA Network Open.*
